# Cobalt–metalloid alloys for electrochemical oxidation of 5-hydroxymethylfurfural as an alternative anode reaction in lieu of oxygen evolution during water splitting

**DOI:** 10.3762/bjoc.14.121

**Published:** 2018-06-13

**Authors:** Jonas Weidner, Stefan Barwe, Kirill Sliozberg, Stefan Piontek, Justus Masa, Ulf-Peter Apfel, Wolfgang Schuhmann

**Affiliations:** 1 Analytical Chemistry – Center for Electrochemical Sciences (CES), Ruhr-Universität Bochum, Universitätsstraße 150, D-44780 Bochum, Germany; 2 Anorganische Chemie I, Ruhr-Universität Bochum, Universitätsstraße 150, D-44780 Bochum, Germany; 3 Fraunhofer UMSICHT, Osterfelder Straße 3, D-46047 Oberhausen, Germany

**Keywords:** alternative anode reaction, electrocatalysis, electrosynthesis, HMF oxidation, hydrogen evolution reaction

## Abstract

The electrochemical water splitting commonly involves the cathodic hydrogen and anodic oxygen evolution reactions (OER). The oxygen evolution reaction is more energetically demanding and kinetically sluggish and represents the bottleneck for a commercial competitiveness of electrochemical hydrogen production from water. Moreover, oxygen is essentially a waste product of low commercial value since the primary interest is to convert electrical energy into hydrogen as a storable energy carrier. We report on the anodic oxidation of 5-hydroxymethylfurfural (HMF) to afford the more valuable product 2,5-furandicarboxylic acid (FDCA) as a suitable alternative to the oxygen evolution reaction. Notably, HMF oxidation is thermodynamically more favorable than water oxidation and hence leads to an overall improved energy efficiency for H_2_ production. In addition, contrary to the “waste product O_2_”, FDCA can be further utilized, e.g., for production of polyethylene 2,5-furandicarboxylate (PEF), a sustainable polymer analog to polyethylene terephthalate (PET) and thus represents a valuable product for the chemical industry with potential large scale use. Various cobalt–metalloid alloys (CoX; X = B, Si, P, Te, As) were investigated as potential catalysts for HMF oxidation. In this series, CoB required 180 mV less overpotential to reach a current density of 55 mA cm^−2^ relative to OER with the same electrode. Electrolysis of HMF using a CoB modified nickel foam electrode at 1.45 V vs RHE achieved close to 100% selective conversion of HMF to FDCA at 100% faradaic efficiency.

## Introduction

Energy production from renewable sources continues to contribute to a significant growing share of current and future energy requirements. However, the intermittency of renewable energy sources renders it a necessity to develop new technologies to convert and store surplus energy, which can be made accessible on demand [1]. Energy storage in hydrogen as a highly versatile energy carrier, which can be inexhaustibly obtained from water, is very appealing [2]. For the conversion of renewable energy into storable hydrogen, electrochemical water splitting turns out to be among the most promising approaches. However, this reaction is energy intensive, especially due to sluggish kinetics and high overpotential of the oxygen evolution reaction (OER) leading to a low energy conversion efficiency [3].

**Scheme 1:**
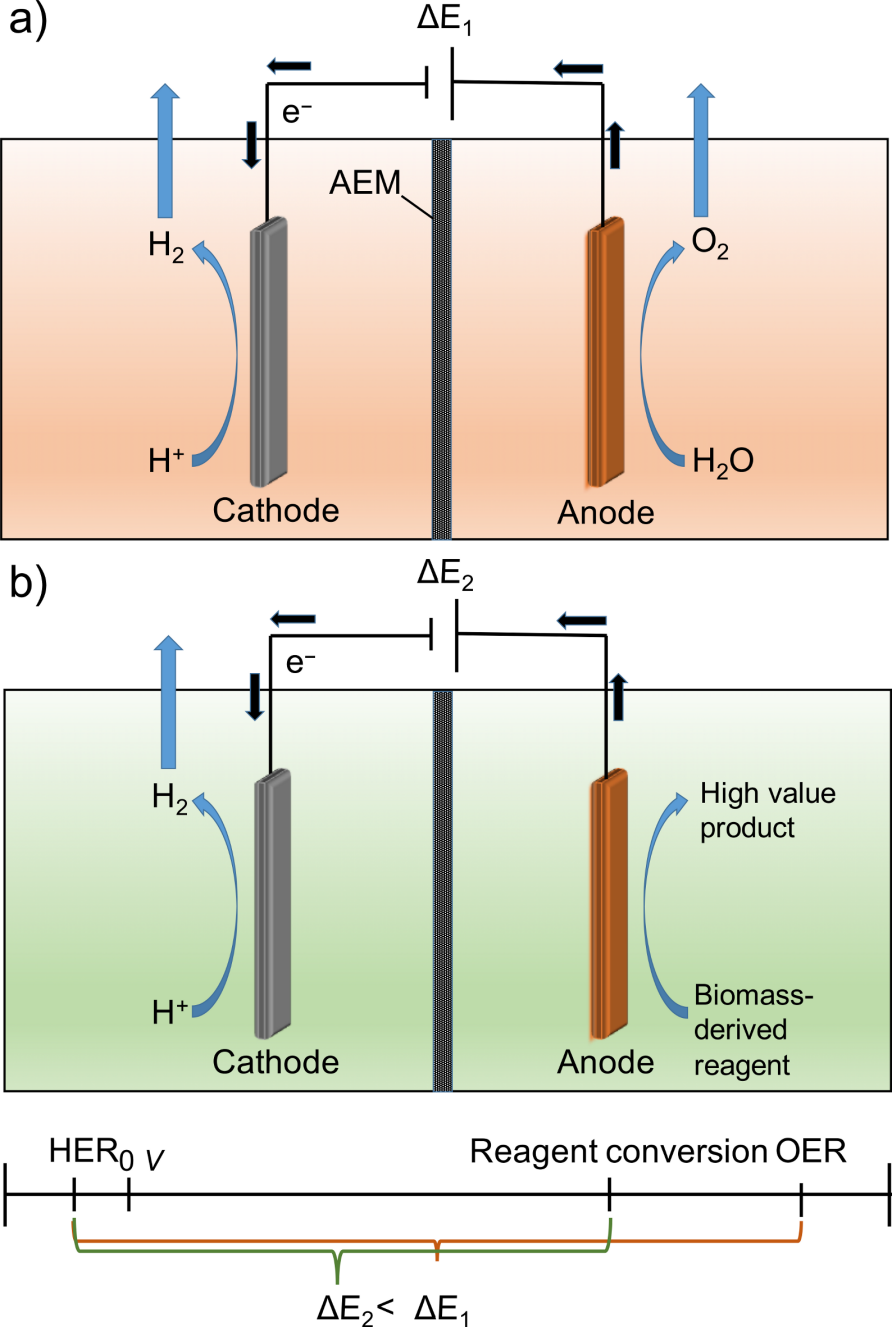
Conventional water electrolyzer (a) and electrolyzer using an alternative anode reaction (b) in alkaline media with an anion exchange membrane (AEM) including a comparison of the expected cell voltages (Δ*E*).

Importantly, the oxygen that is produced as an inevitable byproduct possesses comparatively low economic value with respect to the energy demand of its production. Thus, replacing the OER by a thermodynamically and/or kinetically more favorable anode reaction is desirable in order to increase the energy efficiency of the hydrogen production and hence to facilitate the development of large scale electrochemical hydrogen production. Advantageously, the oxidation of an alternative substrate at the anode, for example a biomass-derived fuel, allows to generate high value products besides hydrogen concomitantly with an increase in energy conversion efficiency during electrolysis [4]. Alternative anode products replacing oxygen evolution could be produced in new generation electrolyzers (see Scheme 1) [5].

According to the report of the US Department of Energy from 2004, the fructose-derived hydroxymethylfurfural (HMF) and its oxidation product 2,5-furandicarboxylic acid (FDCA) are bio-refinery based chemicals for a “green” chemical industry [6–8]. FDCA was suggested to replace, e.g., terephthalic acid as building block for the formation of polyamides, polyesters, and polyurethanes [6]. Especially, the polymer polyethylene terephthalate (PET), a perpetually used polymer, could be substituted by FDCA-based polyethylene 2,5-furandicarboxylate (PEF) produced via a green chemical synthetic route. Thus, HMF oxidation would lead to a product of high economic value and ecological relevance [9].

Classically, the oxidation of HMF is performed by means of homogeneous and heterogeneous catalysis [10–11]. The conversion of HMF to FDCA via homogeneous catalysis, however, suffers from two main drawbacks. Firstly, the yield in FDCA is relatively low due to poor oxidation selectivity. Secondly, the recycling of the catalysts and the purification of FDCA from the reaction mixtures is time-consuming and costly. Heterogeneous catalysts are more easily separated from the reaction solution and can therefore be reused for further oxidation of HMF. Noble metal catalysts such as Pt [12–16], Au [16–20], Pd [16,21–23], and Ru [24] are frequently employed as heterogeneous catalysts for the oxidation of HMF. However, the high cost of the noble metal catalysts has aroused interest in transition metal catalysts and alternative methods for the oxidation of HMF. The electrochemical oxidation of HMF to FDCA was first reported in 1991 by Grabowski and co-workers [25]. Here, HMF was selectively converted to FDCA in NaOH (1.0 M) as electrolyte using a nickel oxide/hydroxide anode achieving a yield of 71% [25]. Strasser and Vuyyuru observed the degradation of HMF to humin type products in highly alkaline solutions and proposed a lower working pH value (<13) for electrocatalytic HMF oxidation. However, using a Pt electrode at pH 10, only sluggish FDCA formation in trace amounts was achieved (below 1%) [26]. This result highlights the need for highly efficient catalysts to enhance the oxidation of HMF to FDCA at high pH values. Li and co-workers studied the electrochemical oxidation of HMF on carbon black supported monometallic Pd/C and Au/C, and bimetallic Pd-Au/C catalysts [27]. Their studies revealed that the reaction pathway was influenced by the type of catalyst and the applied potential [27]. Furthermore, Choi and Cha found that the overpotential required to initiate HMF oxidation was considerably decreased by introducing 2,2,6,6-tetramethylpiperidine-1-oxyl (TEMPO) as an electron mediator to the electrolyte [4]. Despite promising results in synthesizing FDCA, this method suffers from the high cost of TEMPO, which had to be added in 1.5 equivalents relative to HMF [4]. The elaborate separation of TEMPO from FDCA appeared to be an additional disadvantage [24]. Recently, Sun and co-workers reported the electrochemical oxidation of HMF using various non-precious cobalt and nickel based bifunctional HER/OER water splitting electrocatalysts, namely CoP on copper foam, Ni_2_P and Ni_3_S_2_ on nickel foam, in a one compartment batch type electrochemical reactor [5,28–29]. We recently reported on the synthesis and application of alloys of cobalt with boron and phosphorus as exceptionally active bifunctional HER/OER catalysts for water splitting. Inspired by these results, we herein report on the application of different cobalt–metalloid alloys (Co–X; X = B, Si, P, As and Te) for the electrochemical HMF oxidation. With the exception of CoP, all tested materials were to the best of our knowledge not described before for the electrochemical HMF oxidation. Screening of the various cobalt–metalloid alloys supported on Ni RDE electrodes revealed CoB to be the most efficient HMF oxidation catalyst. Using a CoB modified Ni foam as anode material, and Ni foam as the cathode in a continuous flow reactor with an anion exchange membrane separating the anodic and cathodic compartments, a faradaic efficiency of 100% for HMF oxidation, 100% selectivity to FDCA with a yield of 94% was observed.

## Results and Discussion

### Catalyst screening

**Figure 1:**
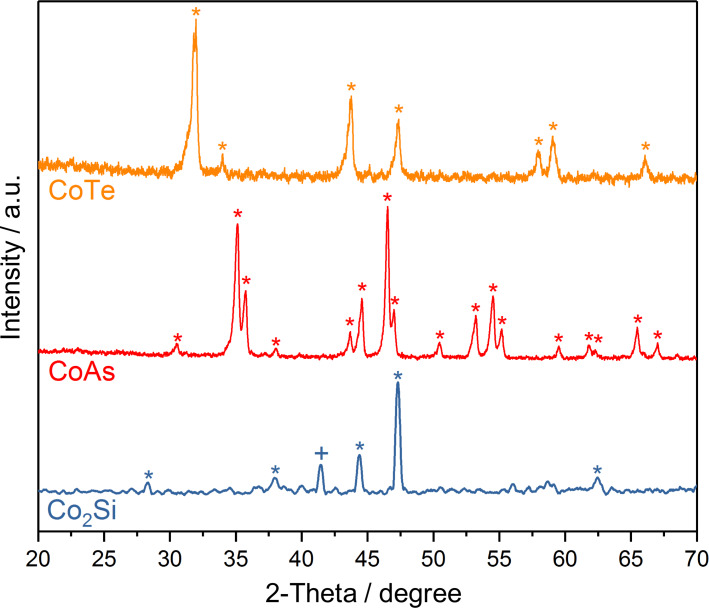
XRD patterns of Co_2_Si, CoTe, CoAs. Cobalt is indicated by (+) and signals corresponding to the desired materials are indicated by (*).

Replacing the O_2_ evolution reaction with an alternative energetically less demanding and more facile process leading to a more valuable product is an appealing approach for increasing the competitiveness of the electrochemical hydrogen production. 5-Hydroxymethylfurfural (HMF) is a biomass-derived compound that can be converted to economically more valuable 2,5-furandicarboxylic acid (FDCA) via electrochemical oxidation at a comparatively lower potential than that required for water oxidation. Suitable catalysts are, however, required to achieve a selective oxidation of HMF to FDCA with a high conversion yield. We recently demonstrated that the modification of cobalt with boron and phosphorus alters the electronic and lattice properties of elemental cobalt leading to significant enhancement of its activity for the oxygen evolution reaction (OER) and the hydrogen evolution reaction (HER). We therefore envisioned that modification of cobalt with other metalloid elements, that is, cobalt–metalloid alloys (CoX; X = B, Si, P, As and Te), could likewise lead to an enhanced electrocatalysis for reactions other than the OER and the HER. To this end, alloys of cobalt with boron, silicon, phosphorus, arsenic and tellurium were screened for their electrocatalytic activity for HMF oxidation. A detailed synthetic procedure to afford cobalt boride (CoB) and cobalt phosphide (CoP) was published previously together with a detailed material characterization [30–33]. CoAs as well as CoTe was synthesized at 1100 °C from the elements in sealed glass ampules. In contrast to CoAs and CoTe, Co_2_Si was not accessible via a similar high-temperature synthesis from the elements and was obtained by salt metathesis from Mg_2_Si and CoCl_2_ at 400 °C under inert conditions. Characterization of the materials was performed using powder XRD (Figure 1). For Co_2_Si, the observed reflexes correlate well with those observed for PXRD patterns generated from single crystalline Co_2_Si (PDF04-0847) with only little contribution of metallic Co [34]. To further confirm the composition of this material, we performed energy-dispersive X-ray spectroscopy (EDS) to elucidate the structural composition of the material. EDS unequivocally revealed a Co:Si ratio of 2:1 and further supported the formation of Co_2_Si (Supporting Information File 1, Figure S1). In contrast to Co_2_Si, CoAs and CoTe are identified as the phase pure arsenide [35] (ICDD 00-052-0774) and telluride [36] (pdf 00-034-0420), respectively (Figure 1).

**Figure 2:**
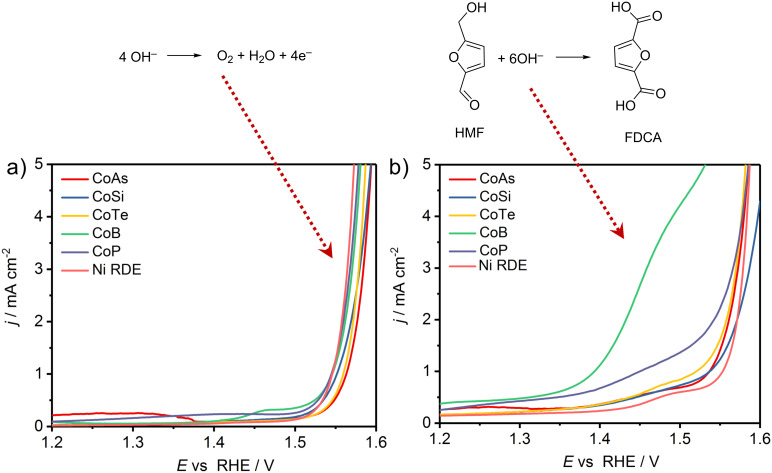
Linear sweep voltammograms of CoP, CoB, CoTe, Co_2_Si, CoAs modified and blank Ni RDEs for the OER (a) and the HMF oxidation (10 mM HMF) (b) (1600 rpm, 1 M KOH, 2 mV s^−1^).

An initial screening of the catalysts with respect to their catalytic HMF oxidation activity was performed by potentiodynamic rotating disk electrode (RDE) voltammetry in 1.0 M KOH, in the absence (Figure 2a) and presence of HMF (10 mM, Figure 2b). Linear sweep voltammograms (LSVs) in the absence of HMF show similar activity toward the OER for all the tested materials. However, there is an apparent contribution of the nickel substrate to the measured OER activity. On the contrary, a pure Ni electrode did not show any significant HMF oxidation in the investigated potential window and hence any increased HMF oxidation current is considered to be due to the activity of the catalyst materials. LSVs recorded in the presence of HMF revealed an increase in the measured current density at substantially lower anodic potentials, and exhibited a plateau-like behavior, which is especially pronounced for CoAs, CoSi and CoTe. In the case of CoB and CoP, the HMF oxidation current merged with the OER current and a shoulder-like feature rather than a plateau was observed. This feature is, however, significantly more pronounced in case of CoB. A comparison of the features shown in Figure 2a and 2b indicates that the OER is negligible in the potential window between 1.2 V to 1.5 V vs RHE, where HMF oxidation is facilitated by all investigated catalyst materials. However, parallel O_2_ evolution at slow rates cannot be fully excluded.

For a comparison of the HMF oxidation activity of the investigated catalysts, two performance criteria were taken into account, namely, the current density achieved at a potential of 1.45 V vs RHE, a potential where the contribution of OER activity is negligibly small, and the potential necessary to attain a current density of 1 mA cm^−2^. The CoB electrocatalyst achieved a current density of 2.69 mA cm^−2^ at 1.45 V and delivered 1.0 mA cm^−2^ at 1.39 V. The overpotential for HMF oxidation is thus decreased by 160 mV as compared to the OER displaying the same current density and using the same electrode. In the series of materials tested, CoB is the most active electrocatalyst for the oxidation of HMF. A detailed comparison of the activities of all investigated CoX-based catalysts (X = B, Si, P, As and Te) is highlighted in Table 1.

**Table 1:** A comparison of the OER and HMF oxidation activity of CoB, CoP, CoAs, Co_2_Si and CoTe modified Ni RDEs (data taken from LSVs of Figure 2).

	Current density [mA cm^−2^] @ 1.45 V vs RHE (no HMF)	Current density [mA cm^−2^] @ 1.45 V vs RHE (10 mM HMF)	Potential [vs RHE, V] @ 1 mA cm^−2^ (no HMF)	Potential [vs RHE, V] @ 1 mA cm^−2^ (10 mM HMF)

CoAs	0.11	0.54	1.56	1.54
Co_2_Si	0.13	0.52	1.55	1.54
CoTe	0.10	0.57	1.56	1.52
CoB	0.26	2.69	1.55	1.39
CoP	0.23	1.00	1.54	1.45
Ni electrode	0.08	0.37	1.55	1.55

### HMF oxidation in a continuous flow reactor

The surprisingly high HMF oxidation activity of CoB made it especially interesting to further study the product distribution and FDCA yield in a continuous mode. For this, a flow reactor was employed which contains two nickel foam (NF) electrodes separated by an anion exchange membrane (Supporting Information File 1, Figure S2). The NF anode (1 cm × 1 cm) was modified with CoB by means of spray coating while pure NF was used as cathode material. The photographs in Figure S3 (Supporting Information File 1) show the contrast of the bare NF and the CoB-modified NF, while Figure S4 (Supporting Information File 1) shows scanning electron (SEM) micrographs of CoB-modified NF.

**Figure 3:**
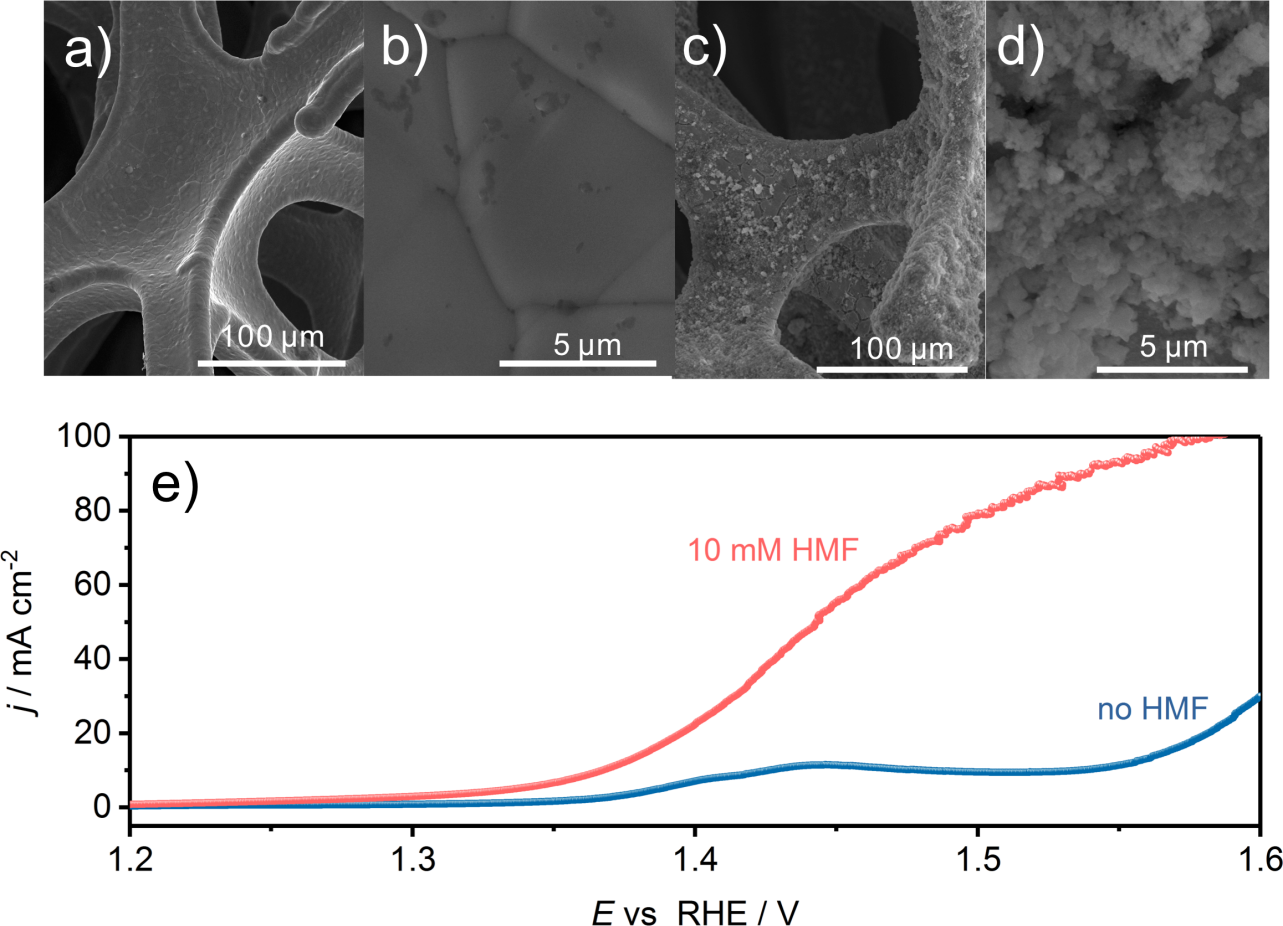
SEM micrographs of bare (a, b) and CoB-modified (c, d) NF with 1000× or 20000× magnification, respectively. e) LSVs of CoB modified NF in the absence and presence of HMF (10 mM) in the flow reactor (1 M KOH, 2 mV s^−1^, 18 mL min^−1^).

The initially smooth NF surface (Figure 3a,b) appears rough with nanometer sized agglomerates of CoB nanoparticles after the spray-coating process (Figure 3c,d; Supporting Information File 1, Figure S4). The current density (normalized to the geometric area of NF) at 1.45 V vs RHE reached 55 mA cm^−2^ during the oxidation of HMF, while a 180 mV more anodic potential was necessary to achieve the same current density during OER in the absence of HMF (Figure 3e). The LSV recorded in the absence of HMF shows a pre-OER oxidation peak at around 1.40 V to 1.45 V vs RHE originating from a convolution of Ni and Co oxidation processes from the NF substrate and from the catalyst, respectively [32]. Evidently, as already seen during RDE voltammetry the presence of HMF allowed an oxidation process to occur prior to the OER.

However, neither the degree of oxidation, product distribution nor the reaction pathway for HMF oxidation can be resolved from voltammograms. Constant potential electrolysis at 1.45 V vs RHE was performed and product analysis at various time points during electrolysis was conducted by means of high-performance liquid chromatography (HPLC) to monitor the oxidation of HMF to FDCA. The current density vs time transient (Supporting Information File 1, Figure S5) shows a rapid decrease of the measured current density within the first minutes, approaching zero current after approximately 1 h, indicating complete HMF conversion. The consumed charge shows a corresponding steep rise with a change in the slope after about 40 min of electrolysis. The continuous increase of charge after a steady current associated with HMF oxidation was attained is attributed to the underlying OER that already proceeds at 1.45 V vs RHE, however, at a very low reaction rate. Complete (100%) HMF conversion requires 6 Faradays or 58 C for 10 mL of a 10 mM HMF solution. In this case, 58.8 C or 6.1 Faradays were transferred after 60 min of electrolysis further pointing towards complete conversion of HMF.

### High-performance liquid chromatography product analysis

**Figure 4:**
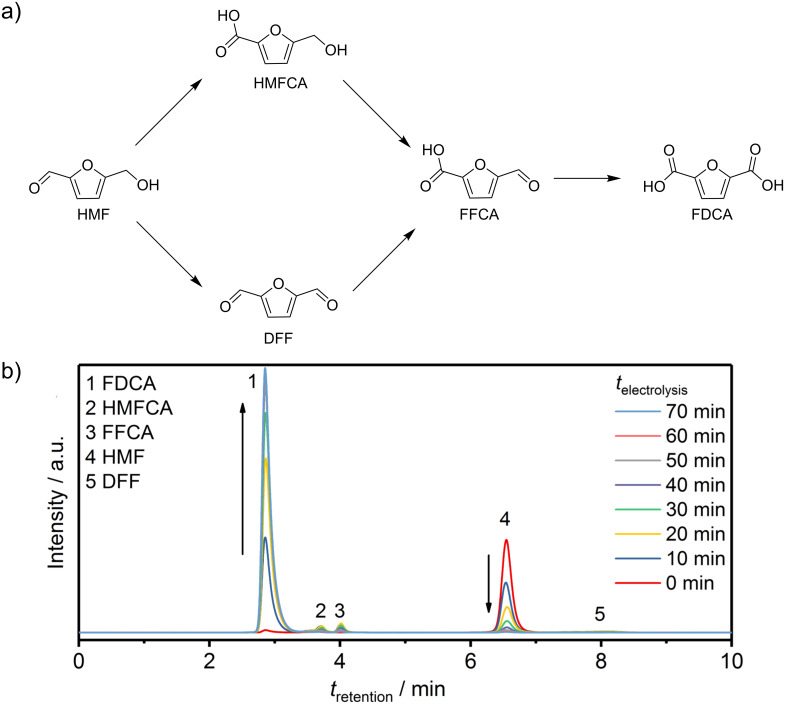
a) Reaction pathways of HMF oxidation; b) chromatograms at various times during constant potential electrolysis at 1.45 V vs RHE.

HPLC was employed to qualitatively and quantitatively determine the conversion of HMF and all potential side products. HMF oxidation starts with oxidation of either the alcohol or the aldehyde leading to the dialdehyde 2,5-diformylfuran (DFF) or to 5-hydroxymethyl-2-furancarboxylic acid (HMFCA), respectively (Figure 4a). Subsequent oxidation of DFF and HMFCA leads to 5-formyl-2-furancarboxylic acid (FFCA) and finally to FDCA. The chromatograms revealed signal changes especially at retention times of 2.87 and 6.54 min, which correspond to FDCA and HMF, respectively. The intensity of the HMF signal at 6.54 min decreased gradually with time until it disappeared finally after 70 min of electrolysis, indicating complete oxidation of HMF. Correspondingly, the FDCA signal at 2.87 min increased steadily with time reaching a steady state after 60 min of electrolysis. Minor signals from HMFCA and FFCA were observed at retention times of 3.69 and 3.95 min, respectively, while DFF with a retention time of 7.82 min could only be observed during the first 30 min of HMF electrolysis as a very small peak (Figure 4b).

**Figure 5:**
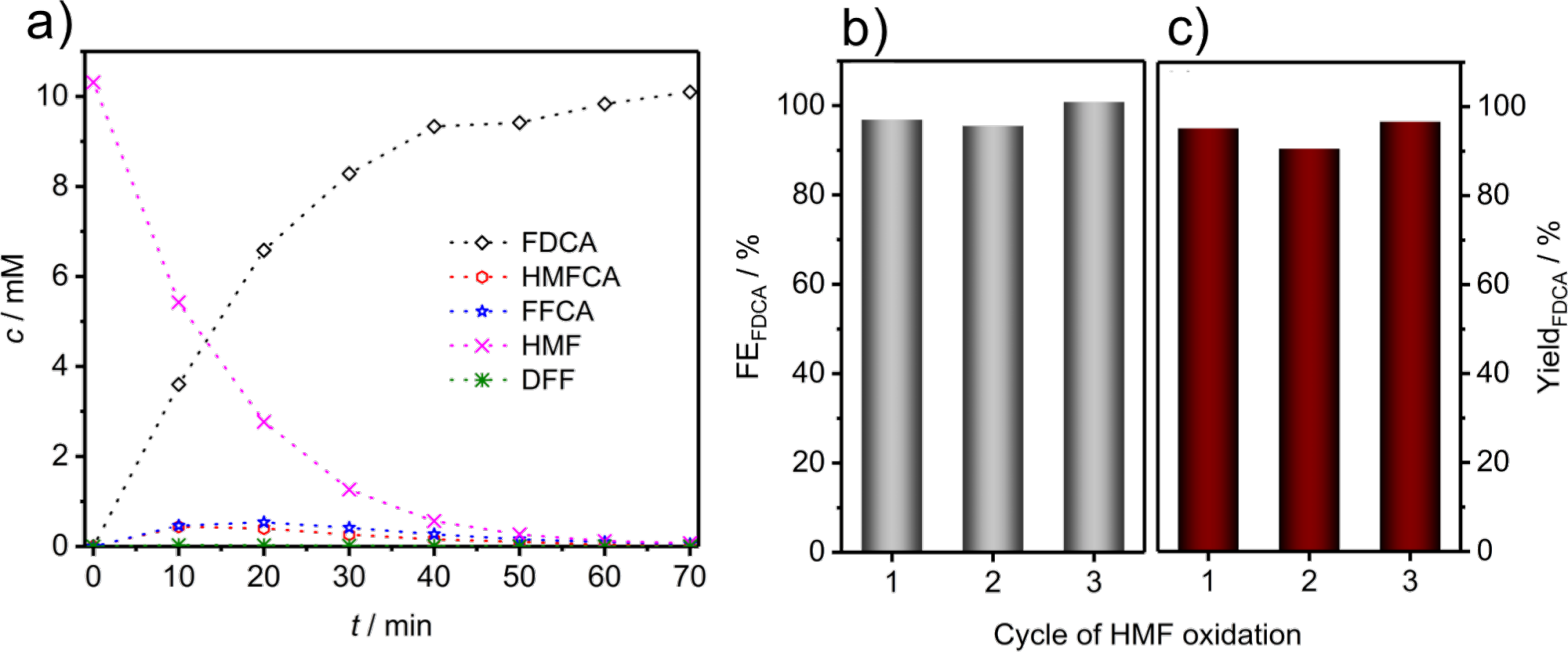
Concentration vs time curve for HMF, HMFCA, DFF, FFCA and FDCA (a); bar diagram of the faradaic efficiency (b) with respect to FDCA formation, and (c) FDCA yield during three consecutive electrolyzes.

Besides qualitative observation of the products and intermediates of HMF oxidation, HPLC was employed for quantification of the intermediates and products at various electrolysis times (Figure 5a). For this, calibration was performed using standard solutions of pure HMF, FDCA and the reaction intermediates (for further information see Supporting Information File 1, Figure S7). The results revealed complete conversion of HMF to FDCA with a faradaic efficiency of 98 ± 2%, thus confirming that the OER is negligible during electrochemical oxidation of the HMF at 1.45 V. According to our HPLC results, HMFCA was the more pronounced intermediate as compared to DFF, which could be due to rapid transformation of DFF to FFCA or slow formation of DFF. Nevertheless, the results clearly indicate that the oxidation of HMF proceeds via both possible pathways forming DFF as well as HMFCA as intermediates, which are then further oxidized to FFCA and finally FDCA. The yield of FDCA was determined to be 94 ± 3% for three consecutive electrolysis cycles, although the faradaic efficiency and thus the selectivity was close to 100%. HMF is known to decompose into humin type structures at a pH value higher than 12 [26]. Nevertheless, a high pH value is necessary to accelerate HMF conversion [37]. HPLC analysis of 10 mM HMF in 1 M KOH without any applied potential revealed an about 10%/h degradation of HMF into electrochemically inactive humins (Supporting Information File 1, Figure S8), which can then obviously not be transformed into FDCA. This explains the determined faradaic efficiency of 98 ± 2% with a yield of 94 ± 3% for the conversion of the fraction of HMF which was not decomposed at the high pH value. Importantly, the HMF degradation rate decreased under electrochemical HMF oxidation conditions. The catalyst modified electrodes showed a high stability and could be used for multiple successive electrolysis cycles with a reproducible HMF to FDCA conversion (Figure 5b,c; Supporting Information File 1, Figure S9). In each cycle, HMF was fully converted to FDCA with a faradaic efficiency of close to 100%. Unlike the OER, the oxidation of HMF does not lead to bubble formation and thus does not induce high physical stress on the catalyst coating. Therefore, the catalyst coating stays intact even after several cycles of HMF electrolysis (Supporting Information File 1, Figure S10). In conclusion, HMF oxidation is not only more energy efficient than the OER but the employed electrodes and catalyst films certainly suffer less deactivation.

## Conclusion

An alternative, energetically less demanding anode reaction forming a more valuable product in lieu of the oxygen evolution reaction is presented. The oxidation of the bio-refinery product HMF in a flow reactor led to selective formation of the corresponding dicarboxylic acid FDCA with a faradaic efficiency of close to 100%. FDCA is an industrially relevant chemical that can be used for the production of polymers with a potential large-scale application. Thus, HMF oxidation at the anode as a complementary reaction to cathodic hydrogen evolution does not only form a product of added value but also leads to a significant decrease of the overpotential necessary to achieve a certain current density as compared to the OER. While HMF tends to decompose in highly alkaline solutions, its electrochemical oxidation is kinetically more favorable and leads to a competition between HMF decomposition and its transformation into FDCA. However, although 10% of HMF decompose within 1 h when stored in 1 M KOH, electrolysis of a 10 mM solution of HMF in 1 M KOH employing CoB as electrocatalyst suppressed this decomposition and afforded high FDCA yields of 94 ± 3%. It is therefore evident that the catalyst is capable to selectively oxidize HMF to FDCA with only minor losses due to decomposition. Further research and optimization of the electrocatalyst could enhance the kinetics of the HMF oxidation and minimize its decomposition even further.

## Experimental

All chemicals were of analytical grade and used without further purification. All aqueous solutions were prepared using ultrapure Milli-Q water (SG Water). KOH was purchased from Carl Roth. HMF, DFF, HMFCA, FFCA and FDCA were from Sigma-Aldrich. Pure elements (Co, As, Te) were supplied by Sigma-Aldrich, Merck, and Alfa Aesar with high purities. All high temperature steps were performed in a tubular furnace in 10 mm quartz ampules evacuated to a pressure of 4 × 10^−2^ mbar. Nickel foam (99.5% purity) used as electrode material was purchased from Goodfellow.

### Catalyst preparation

**Cobalt silicide, Co_2_Si.** CoCl_2_ (0.50 g) and Mg_2_Si (0.15 g) were thoroughly mixed and heated to 400 °C. Subsequently, the mixture was hold isothermal at this temperature for 24 h. The mixture was then allowed to slowly cool to room temperature. The obtained solid was then ground to give a fine powder that was washed with water and dried in vacuum.

**Cobalt arsenide, CoAs.** A mixture of cobalt (440 mg) and arsenic (560 mg) was heated to 700 °C. The temperature was hold for 3 h followed by a heating step to 1100 °C with an isothermal step for 20 h. The mixture was subsequently cooled to room temperature to give CoAs.

**Cobalt telluride, CoTe.** Cobalt telluride was synthesized from the elements Co (0.948 g) and tellurium (2.052 g). The ampule was heated to 800 °C followed by an isothermal step for 2 h. The mixture was then heated to 1100 °C and hold for 15 h. Subsequently, the bulk material was cooled down by switching off the furnace. The product was obtained in form of a purple metallic ingot.

### Electrochemical measurements

#### Electrode preparation

Nickel RDEs (Ø = 3 mm) were polished successively with 1 µm and 0.05 µm alumina polishing paste. Polished electrodes were drop coated with a catalyst ink containing 5 mg mL^−1^ solid catalyst material suspended in a water/ethanol (1:1 v/v) mixture. The catalyst suspension was sonicated for 20 min in order to be homogeneous prior to electrode preparation. After drying in air, the final loading of catalyst material on the electrode was 210 µg cm^−2^.

Nickel foam electrodes were modified by means of spray coating. The NF electrodes were cleaned by immersing in concentrated HCl for 5 min prior the spray-coating process. Residual acid was subsequently removed by washing with water, ethanol and acetone. Clean NF was spray-coated from a stirred suspension of catalyst material (2.5 mg mL^−1^) in a water/ethanol (1:1 v/v) mixture. During the spray coating, the NF electrodes were heated to 80 °C in order to facilitate fast solvent evaporation. The final catalyst loading on NF was ≈1 mg cm^−2^_geom._.

#### Rotating disk electrode measurements

Each measurement was conducted with an Autolab III potentiostat/galvanostat (Metrohm) attached to an Autolab rotator (Metrohm). The experiments were performed in a three-electrode configuration using aqueous 1 M KOH as electrolyte. HMF oxidation was performed using a 10 mM HMF in 1 M KOH solution. A Pt mesh served as counter electrode (CE), and a Ag/AgCl/3 M KCl was used as reference electrode (RE). The potential was converted to the reversible hydrogen electrode (RHE) according to Equation 1:

[1]
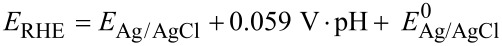


The pH value of the 1 M KOH was determined by means of a pH electrode for high alkaline solutions (Dr. Kornder Anlagen- und Messtechnik, Germany) to be 14.

RDE measurements were performed at a rotation speed of 1600 rpm. In order to correct the potential for the uncompensated electrolyte resistance, electrochemical impedance spectroscopy (EIS) was performed at the open circuit potential at perturbation frequencies between 10 kHz and 200 Hz with an amplitude of 10 mV_pp_. Catalyst conditioning was performed by running 20 cyclic voltammograms between 0 V and 0.5 V vs Ag/AgCl/3 M KCl at a scan rate of 100 mV s^−1^. Linear sweep voltammograms (LSVs) in a potential range between 0 V and 0.7 V vs Ag/AgCl/3 M KCl at a scan rate of 2 mV s^−1^ were performed to determine the HMF oxidation activity.

### Flow reactor measurements

Flow reactor measurements were performed using a VMP-3 potentiostat (Bio-logic) in a specifically designed two compartment flow cell setup shown in Figure S2 (Supporting Information File 1). Two separated electrolyte circuits were used for the anode and the cathode compartment. Experiments were conducted in three-electrode configuration using aqueous 1 M KOH as electrolyte solution containing 10 mM HMF. The WE compartment and the CE compartment were separated by a PEEK reinforced anion exchange membrane (Fumatech). The CE consisted of two stacked unmodified Ni foams (1 cm × 1 cm) and a Hg/HgO/1 M KOH electrode served as RE. Catalyst modified NF (1 cm × 1 cm) was used as the WE.

The potential was converted to the RHE scale according to Equation 2:

[2]
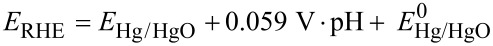


The pH value of the 1 M KOH was determined by means of a pH electrode for high alkaline solutions (Dr. Kornder Anlagen- und Messtechnik, Germany) to be 14.

The electrolyte solution was pumped through the cell with a flow rate of 18 mL min^−1^. Before each measurement an EIS was recorded to determine the uncompensated electrolyte resistance and the potential was corrected accordingly. The conditioning of the catalyst was performed by cyclic voltammetry with 20 cycles between 0.97 V vs RHE and 1.43 V vs RHE with a scan rate of 100 mV s^−1^. The LSVs were measured between 0.97 V vs RHE and 1.65 V vs RHE with a scan rate of 2 mV s^−1^. Constant potential electrolysis was done at 1.45 V vs RHE with a total electrolyte volume of 10 mL in the anode electrolyte reservoir and a HMF concentration of 10 mM. Before and after each electrolysis CVs consisting of one cycle between 0.97 V vs RHE and 1.58 V vs RHE with a scan rate of 2 mV s^−1^ were recorded.

### HPLC analysis

The HPLC system consists of a Knauer pump, a Merck Hitachi L-4250 UV–vis detector, and a Shim-pack GWS C18 column from Shimadzu. Calibration for HMF, HMFCA, DFF, FFCA, and FDCA was conducted with a flow rate of 5 mL min^−1^ using an eluent consisting of 70 vol % 5 mM ammonium formate solution and 30 vol % methanol. The UV detector was recording the absorbance of the different compounds using a single wavelength of 265 nm. Ten μL of sample solutions were diluted with 490 μL of water. A volume of three times the volume of the injection loop (10 µL) was injected.

Conversion of HMF, product yield and faradaic efficiency were calculated according to Equations 3–5, respectively

[3]
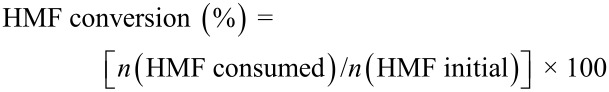


[4]
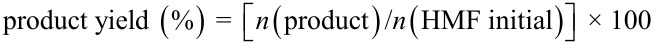


[5]
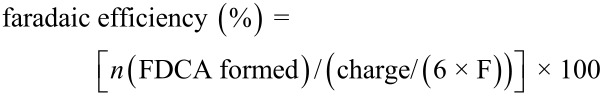


with F being the Faraday constant (96 485 C mol^−1^) and *n* the mol of reactant calculated from the concentration measured by HPLC.

#### HPLC analysis of HMF decomposition

The decomposition of HMF in alkaline solution was measured in a stirred 1 M KOH solution in the presence of 10 mM HMF at 20 °C. Samples were taken directly after the addition of HMF, after 10, 30, 60, 100, and 120 min. The samples were injected into the HPLC system after dilution with 990 μL water.

### Physical characterization

#### X-ray diffractometry

XRD data were obtained using a Panalytical X'PERT Pro MPD X-ray diffractometer with a Cu Kα radiation source (λ = 1.5418 Å) in the 2θ = 20–80° range.

#### Scanning electron microscopy

SEM images were taken using a Quanta ED FEG scanning electron microscope (FEI). The SEM was operated at 20 kV.

## Supporting Information

File 1 Additional figures and chromatograms.
