# Supplementary material for: Cobalt–metalloid alloys for electrochemical oxidation of 5-hydroxymethylfurfural as an alternative anode reaction in lieu of oxygen evolution during water splitting
Source: Beilstein J Org Chem. 2018 Jun 13;14:1436–45. doi: 10.3762/bjoc.14.121 (PMC6009195; doi:10.3762/bjoc.14.121)
Supplement: File 1 — Additional figures and chromatograms. [file Beilstein_J_Org_Chem-14-1436-s001.pdf]

Supporting information

for

Cobalt–metalloid alloys for electrochemical oxidation of 5-hydroxymethylfurfural as an alternative anode reaction in lieu of oxygen evolution during water splitting

Jonas Weidner[1,§], Stefan Barwe[1,§], Kirill Sliozberg[1], Stefan Piontek[2], Justus Masa[1], Ulf-Peter Apfel[2,3] and Wolfgang Schuhmann[1,*]

Address: [1]Analytical Chemistry – Center for Electrochemical Sciences (CES), Ruhr-Universität Bochum, Universitätsstraße 150, D-44780 Bochum, Germany, [2]Anorganische Chemie I, Ruhr-Universität Bochum, Universitätsstraße 150, D-44780 Bochum, Germany and [3]Fraunhofer UMSICHT, Osterfelder Straße 3, D-46047 Oberhausen, Germany

Email: Wolfgang Schuhmann - wolfgang.schuhmann@rub.de

* Corresponding author

[§]Equally contributing authors

Additional figures and chromatograms

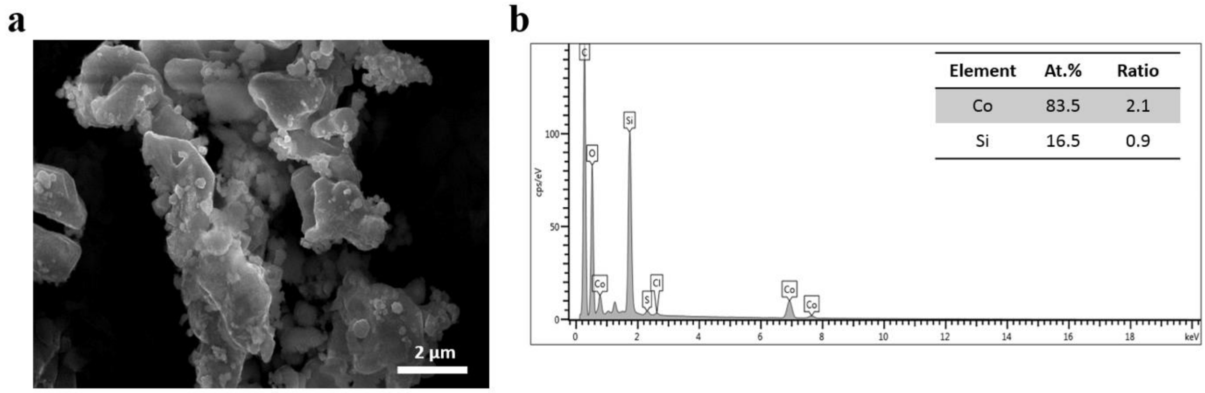

Figure S1: SEM (a) and EDS (b) of Co_2Si.

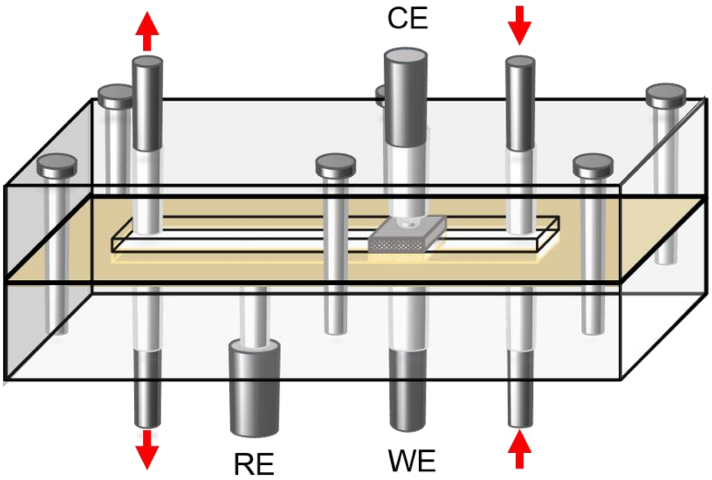

Figure S2: Schematic representation of the flow reactor.

a) b)

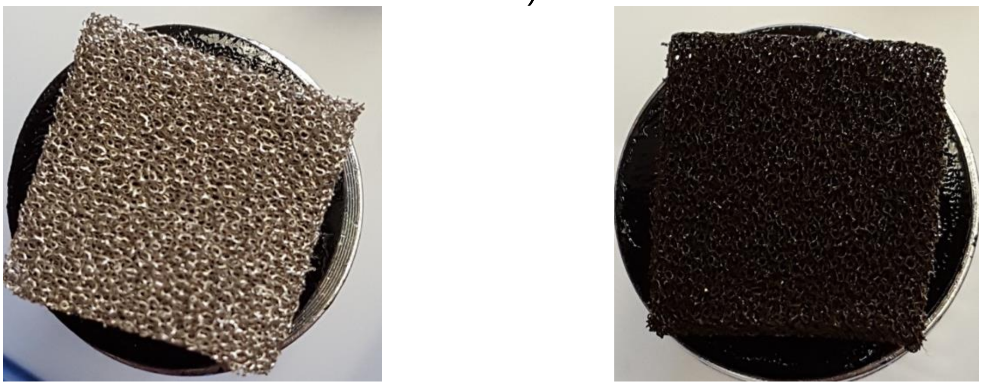

Figure S3: Photographs of bare (a) and CoB-modified (b) nickel foam.

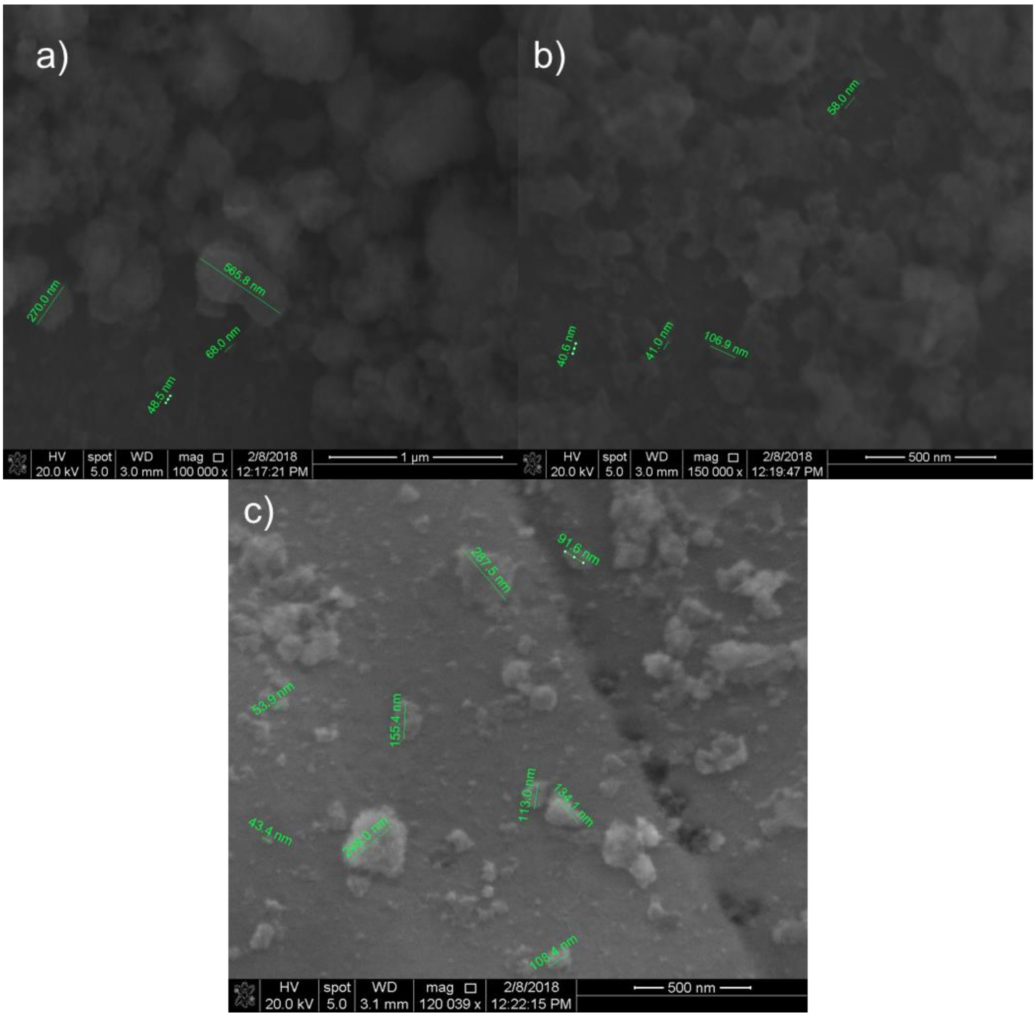

Figure S4: SEM images of CoB-modified nickel foam.

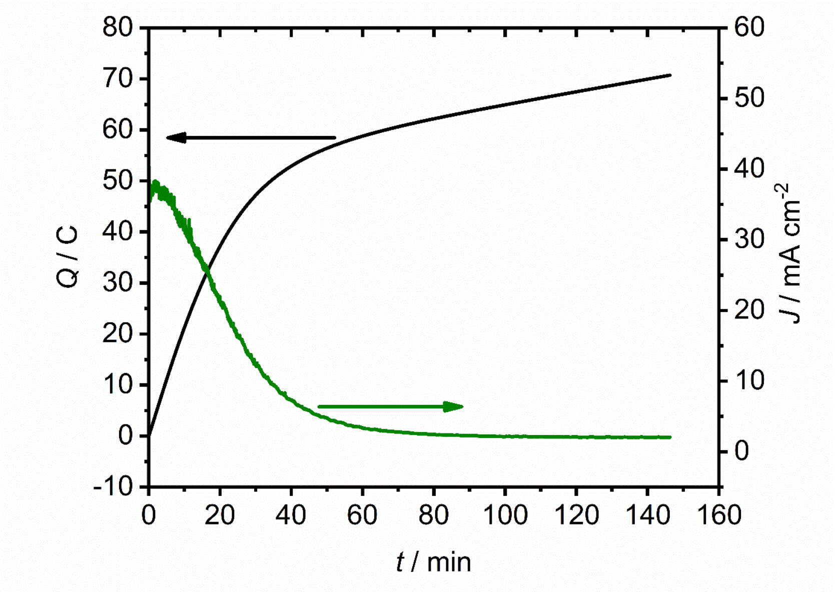

Figure S5: Current vs. time and charge vs. time transients during constant potential HMF electrolysis at an applied potential of 1.45 V vs RHE.

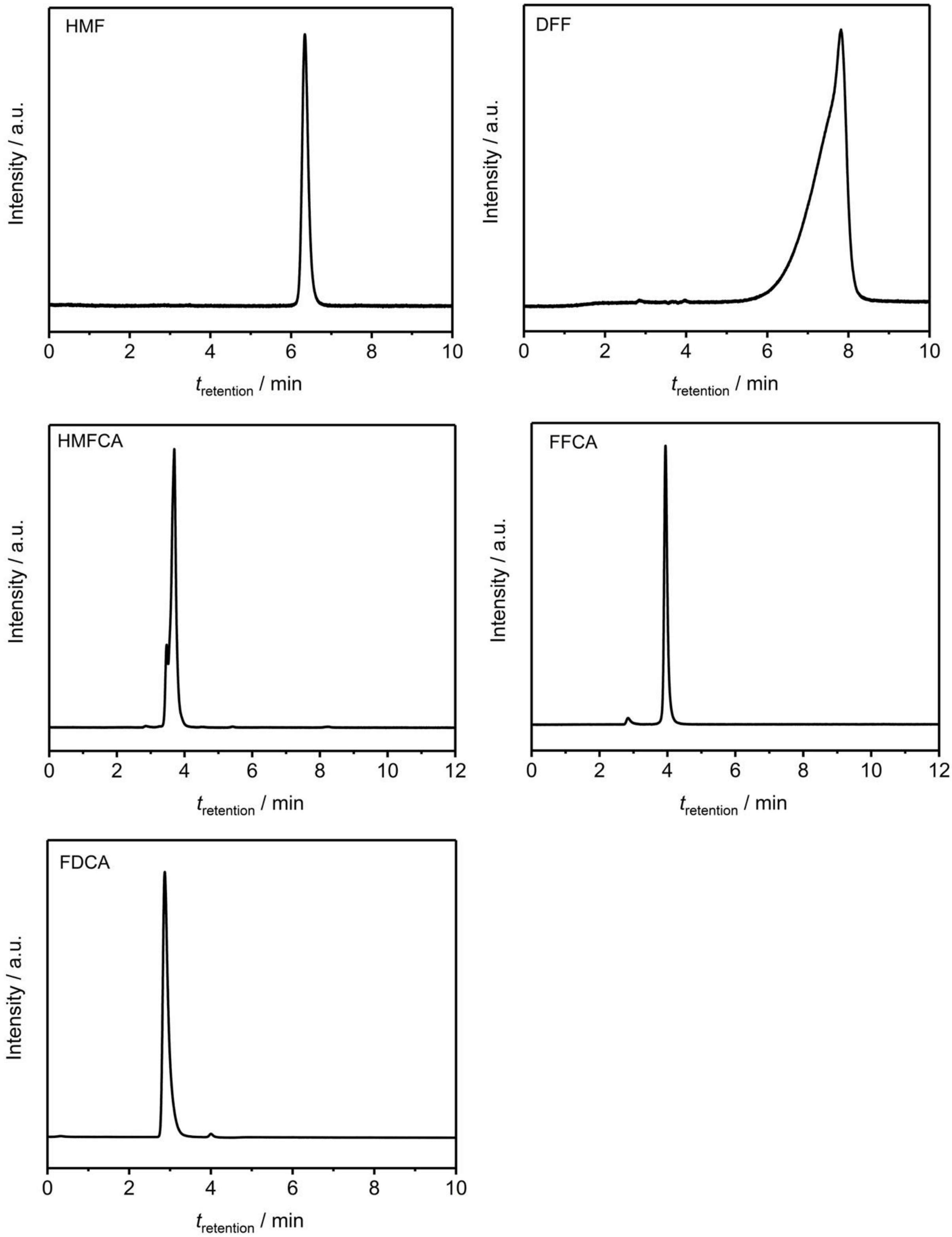

Figure S6: Reference HPLC chromatograms of pure compounds.

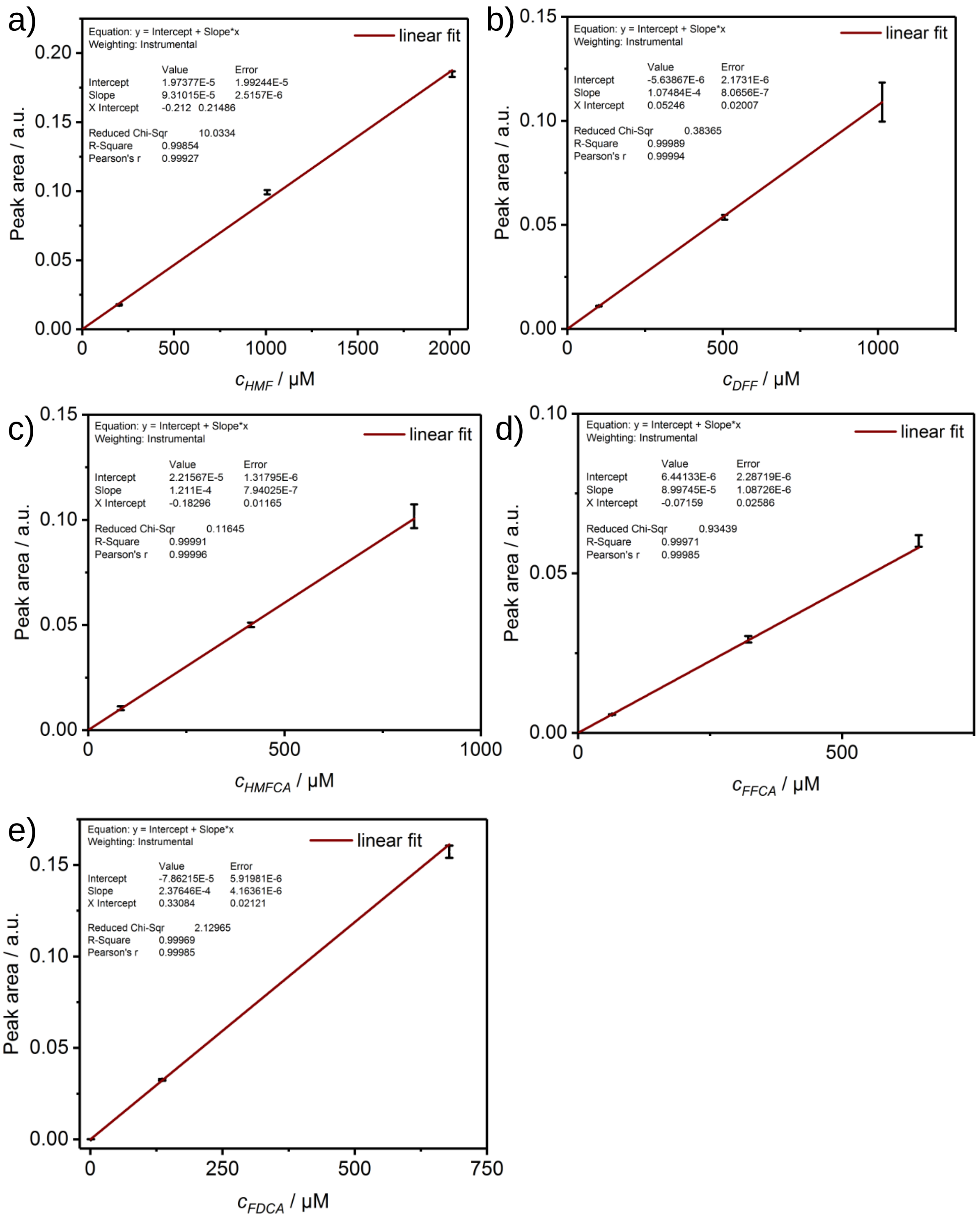

Figure S7: Calibration curves for HMF (a), DFF (b), HMFCA (c), FFCA (d) FDCA (e).

Calibration was done by injecting 10 µL of a solution of the pure compound with a predefined concentration dissolved in 490 µL Milli Q water into the HPLC. The eluent (70 vol % 5 mM ammonium formate and 30 vol % methanol) was pumped with a flow rate of 0.5 mL min^{-1}. For calibration, various compound concentrations were used and the peaks of the corresponding HPLC chromatogram were integrated. Plotting the peak area vs the concentration led to a linear correlation. From the linear fit

equation, the concentrations of the various reactants during electrolysis were calculated.

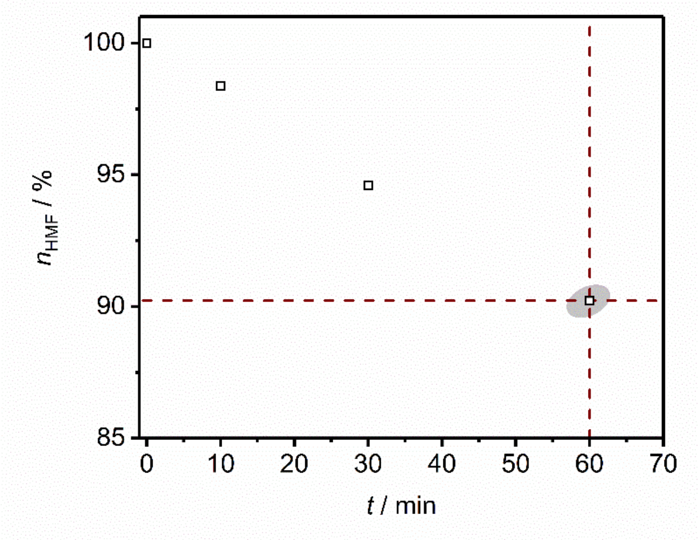

Figure S8: HMF decomposition in 1 M KOH.

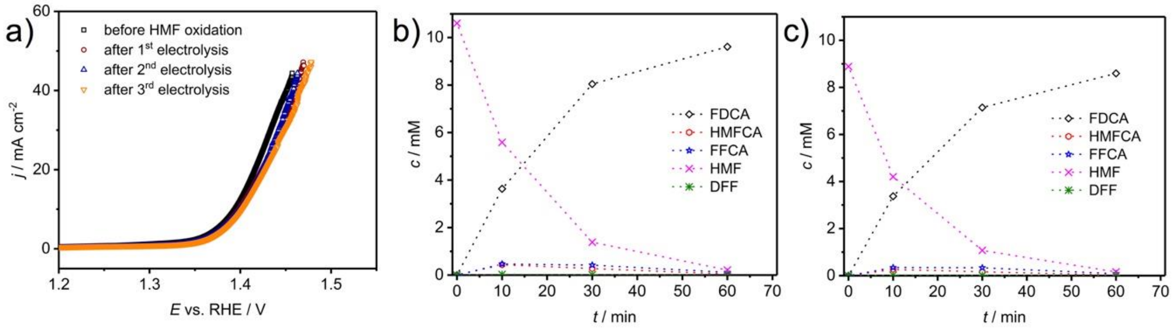

Figure S9: a) LSVs before HMF electrolysis and after each complete HMF oxidation cycle (10 mM HMF in 1 M KOH, 2 mV s^{-1}, 18 mL min^{-1}). Concentration vs time curves for HMF, HMFCA, DFF, FFCA and FDCA of the second (b) and third (c) HMF electrolysis at 1.45 V vs RHE.

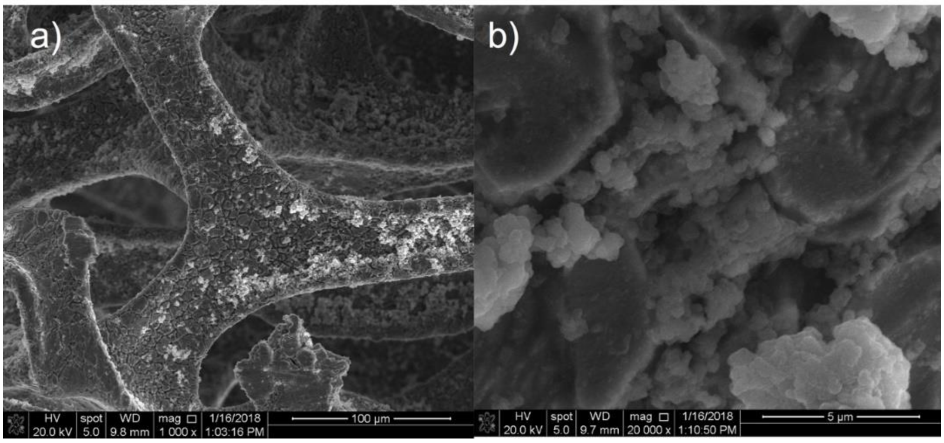

Figure S10: SEM micrographs of the CoB-modified nickel foam electrode after three complete cycles of HMF electrolysis at 1.45 V vs RHE with magnifications of 1000× (a) and 20000× (b).
